# Association between autonomic function, depressive symptoms, and cognitive function in cognitively normal older adults in U.S. POINTER trial

**DOI:** 10.1002/alz.092656

**Published:** 2025-01-09

**Authors:** Hossam A Shaltout, Katelyn R Garcia, Laura D Baker, Heather M Snyder, Kathryn V Papp, Margie J Bailey, Tina E Brinkley

**Affiliations:** ^1^ Wake Forest University School of Medicine, Winston‐Salem, NC USA; ^2^ Wake Forest University, Winston‐Salem, NC USA; ^3^ Alzheimer’s Association, Chicago, IL USA; ^4^ Massachusetts General Hospital, Brigham and Women’s Hospital, Harvard Medical School, Boston, MA USA

## Abstract

**Background:**

Autonomic dysfunction and depression are among the most common causes of disability in high‐income countries and depression has been associated with deficits in various cognitive domains. The degree to which depression associates with autonomic function and modifies risk for Alzheimer’s disease (AD) in older adults is unknown. We investigated the association between depression status and autonomic function measures in 398 participants enrolled in the U.S. POINTER trial (mean age: 68.0±5.3 years, 61% female, 32% people of color).

**Methods:**

Continuous blood pressure and ECG recordings were used to assess baroreflex sensitivity (BRS), blood pressure variability (BPV), heart rate variability as measured by root mean square of successive differences (rMSSD) and standard deviation of beat to beat interval (SDNN), and sympathovagal balance (LF/HF ratio). Participants were classified as depressed if their geriatric depression scale (GDS) score was >4 or if they used antidepressants, or as non‐depressed if their GDS score was ≤4. Autonomic variables were compared according to depression status. Linear regression was used to examine cross‐sectional associations between the outcomes of interest adjusting for age, sex, race/ethnicity, education, and site.

**Results:**

The prevalence of depression was 27% (n = 110). Depressed individuals were more likely to be female (p = 0.0021), have higher cholesterol levels (p = 0.0056), and have lower BRS (p = 0.0016), rMSSD (p = 0.009) and SDNN (p = 0.0003), with a trend for higher LF/HF ratio (p = 0.12). GDS score was negatively associated with BRS (p<0.05), rMSSD (p = 0.04), and SDNN (p = 0.008). Meanwhile, LF/HF ratio positively correlated with measures of global cognition (p = 0.0045), executive function (p = 0.003), and processing speed (p = 0.008), while rMSSD and SDNN positively correlated with the mini‐mental state examination score. SDNN positively correlated with global cognition and executive function (p<0.05). Adjusting for GDS score did not alter the association between autonomic function and cognition.

**Conclusions:**

In this population of older adults at risk for AD, measures of autonomic function were positively associated with cognitive function and negatively associated with depression. Further studies are needed to assess if early diagnosis and treatment of depression in this population could delay the development of AD.

